# Intraoperative magnetic resonance imaging in glioma surgery: a single-center experience

**DOI:** 10.1007/s11060-024-04660-z

**Published:** 2024-04-03

**Authors:** Leyla Mirzayeva, Murat Uçar, Ahmet Memduh Kaymaz, Esra Temel

**Affiliations:** Gazi University, Faculty of Medicine, Department of Radiology, Ankara, Turkey

**Keywords:** Neurosurgery, Glioma, Neuroradiology, Intraoperative Magnetic Resonance Imaging, Progression Free Survival

## Abstract

**Purpose:**

To investigate the effect of intraoperative magnetic resonance imaging (Io MRI) on overall and progression-free survival (OS and PFS), on the extent of resection (EOR) in patients with glioma, and impact of the radiological diagnosis on the decision to continue the surgery when a residual mass was detected on Io MRI.

**Methods:**

The study comprised 153 glioma patients who received surgical treatment between 2013 and 2023. One**-**hundred twenty**-**five of them had Io MRI guidance during surgery. The remainder 28 patients constituted the control group who did not undergo Io MRI. All patients' age at surgery, gender, initial radiological diagnosis, primary tumor localization, EOR, last histopathological diagnosis, and the follow-up periods were recorded.

**Results:**

The rate of tumor recurrence in Io MRI cases was significantly lower compared to the cases in the control group (*p* < .0001). It was decided to continue the operation in 45 Io MRI applied cases. This raised the gross total resection (GTR) rate from 33.6% to 49.6% in the Io MRI group. The frequency of GTR was significantly higher in patients with an initial radiological diagnosis of low grade glioma than those with high grade glioma. The shortest OS was seen in occipital gliomas.

**Conclusion:**

In this study, the convenience provided by the high-field MRI device was explored and proven both in reducing the tumor burden, increasing the PFS, and providing the surgeon with a maximal resection in the first operation.

## Introduction

Although gliomas are rare tumors compared to other malignancies, they cause important symptoms such as focal neurological deficit, seizure, and neurocognitive disorder due to rapid and infiltrative growing pattern [1, 2]. Different modern technologies have been developed for neurosurgeons to achieve the widest possible tumor resection [1]. The distinction between tumor and normal brain tissue is one of the main challenges in achieving the maximum extent of resection (EOR) without causing damage to the functional brain parenchyma. This distinction is more difficult in primary brain tumors which show a diffuse growth pattern resulting in a transitional zone at the tumor margins where both malignant and normal tissue cells are mixed [3].

Neuronavigation and intraoperative magnetic resonance imaging (Io MRI) are superior to other technologies such as Io ultrasound (US), Io computed tomography, and 5-aminolevulinic acid (5-ALA) in distinguishing tumor tissue from normal brain parenchyma due to their high tissue resolution [4, 5]. However, IO MRI is not widely available in many centers where ultrasound and 5-ALA are quite common. In addition to providing real-time information about EOR, Io MRI also allows updating of the anatomical changes that occur as a result of tumor resection, cerebrospinal fluid evacuation, and shift with the neuronavigation system [6, 7].

Io MRI has been shown to be associated with a higher rate of progression-free survival (PFS) in gliomas and pituitary adenomas compared to conventional neuronavigation [3, 8]. In this article, we present our 10-year experience with patients who underwent Io MRI for glioma surgery. The primary aim of the study was to investigate the effect of Io MRI on EOR, overall survival (OS), and PFS time in patients with glioma during the 10-year follow-up period. We also compared preoperative MRI reports with final histological results to examine the concordance between radiological and pathological diagnoses. Another research focus was the impact of the preoperative radiological diagnosis on the decision to continue the surgery when a residual mass was detected on the Io MRI.

## Materials and methods

### Study design

Institutional Clinical Research Ethics Committee approval (registration number: 99) was obtained for data collection. We retrospectively reviewed 300 adult patients who underwent glioma surgery in Gazi University Faculty of Medicine Department of Neurosurgery between January 2013 and January 2023. All study procedures were in accordance with the principles outlined in the Helsinki Declaration. Patients with a diagnosis other than glioma (n = 78), patients who had undergone previous surgery (n = 28), patients whose preoperative MRI data could not be obtained (n = 10), patients whose postoperative follow**-**up data could not be obtained (n = 26), and patients who underwent surgery for diagnostic biopsy (n = 5) were excluded from the study. Occasionally, complications may arise during surgery; for example, the patient may have seizures when the surgeon attempts an extensive tumor resection. In some cases, during Io cortical-subcortical mapping, it may be revealed that the tumor has infiltrated vital functional areas (motor, linguistic, tactile, visual, and cognitive), especially in high grade gliomas (HGG). In this case, although the surgeon starts the operation with the intention of removing a large part of the tumor, this cannot be achieved. If the surgeon was only able to remove less than 5% of the tumor volume, then the tumor resection rate is included in the "biopsy" category according to the classification [9]. While extensive resection was planned in 4 cases in the Io MRI group and 3 cases in the control group, less than 5% of the tumor volume could be excised due to the risk of permanent neurological disfunction. This situation is different from diagnostic biopsy.

**Fig. 1:**
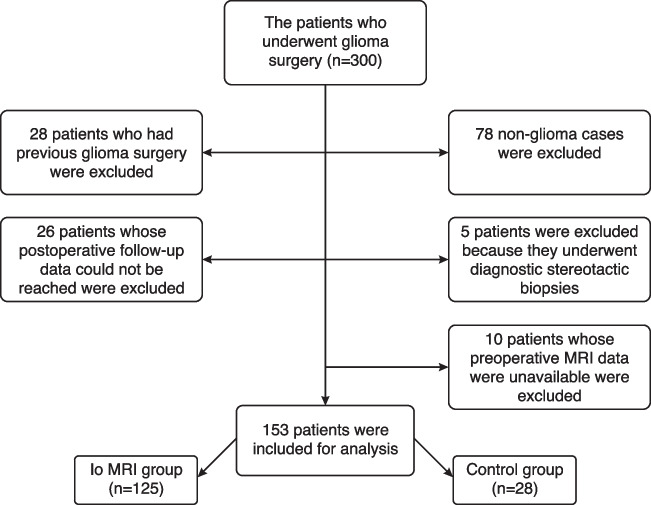
A flowchart showing the exclusion criteria in the study

Overall, 153 patients were included in this study (Fig. 1). All patients signed informed consent form. One-hundred twenty**-**five of 153 patients (81.7%) underwent Io MRI. The remaining 28 (18.3%) patients formed the control group.

One day before the surgery, neuronavigation MRI was performed in all patients with the appropriate protocol depending on the enhancement character of the lesion (after gadolinium injection 3D T1 MPRAGE or 3D FLAIR with 1**-**mm slice thickness). Postoperative MRI was done for both groups within 48 h of surgery. Patients' age at the surgery, gender, initial radiological diagnosis, primary tumor localization, EOR, final histopathological diagnosis, Ki-67 index, and follow-up were recorded.

## Io MRI group

One**-**hundred twenty**-**five patients had surgery in the operating theater with a 3 Tesla MRI device (Siemens Health Care Magnetom Verio, Erlangen, Germany) in the adjacent room. After general anesthesia, a neuronavigation system (StealthStation® Medtronic® Inc, Minneapolis, USA) was set up for all patients. Regarding Io MRI sequences, a 3D T1W volumetric sequence was performed in each patient to readjust neuronavigation. MRI sequences included axial T2-weighted FSE sequences (repetition time: 4200, echo time: 107, matrix: 256 × 256, FOV: 220), 3D FLAIR (repetition time: 9000, echo time: 85, matrix: 256 × 256, FOV: 220), axial DWI (b**-**values of 1000 s/mm^2^, repetition time: 4000, echo time: 90, matrix:128 × 128, FOV: 220), and postcontrast T1 MPRAGE (repetition time: 1750, echo time: 2.93, matrix: 250 × 250, FOV: 250). Postcontrast images were obtained by injecting 20 cc of gadoteric acid (Gd-DOTA, Dotarem) at a rate of 5 mL/s through a peripheral arm vein. The main purpose during the surgery was to obtain a maximum resection without causing neurologic sequelae. In patients with contrast**-**enhancing tumors, gross total resection (GTR) was defined as the complete removal of the enhancing tissue. GTR for unenhanced tumor was defined as the disappearance of pathological tissue seen on T2/FLAIR sequences. The EOR was accepted as near total resection, subtotal resection, partial resection, and biopsy if > 95%, ≥ 80%, 79**–**5%, and < 5% of the tissue was removed, respectively [9].

## Control group

Twenty**-**eight patients in the control group underwent craniotomies in a neurosurgery operating room with a neuronavigation system but without the use of Io MRI guidance. Due to technical conditions (technical malfunction or maintenance period of the device) Io MRI could not be performed in the control group. The surgeon did not recommend postponing surgery because of the presence of neurological findings.

## Volumetric analyses

The volume of the tumors was measured manually, using the editor module of 3D-Slicer version 5.0.2, which is a free, open-source medical image computing and visualization software program. The measurement results were recorded in cubic centimeters. EOR was calculated using the following formula: (preoperative tumor volume – postoperative tumor volume)/preoperative tumor volume [10].

## Statistical analyses

Statistical Package for the Social Sciences was used for statistical analysis (version 22.0; SPSS, Inc., Chicago, IL). For continuous variables, the mean ± standard deviation (SD) or median ± interquartile range (IQR) was given, whereas percentages were used for categorical data. The Chi-Square test was used to compare categorical variables between groups. OS was determined by subtracting the date of the first surgery from the date of death, or the most recent date on the medical records. PFS was defined as the time between the date of the first operation and the date of recurrence.

In a study similar to ours published by Olubiyi et al. [11], the hazard ratio (HR) was calculated according to the OS time of the Io MRI group and the control group and was found to be 0.43. With the STATA 17.0 program (a statistical software for data science), the number of people to be included in the study was determined as at least 121 for the Io MRI group and at least 25 for the control group, based on the values of type 1 error: 5%, power: 95%, allocation ratio: 5 and HR 0.43. Accordingly, the number of cases in our study (125 Io MRI group, 28 control group) exceeds the required minimum number. The Kaplan–Meier survival curves were used to assess patient survival in both groups. The Log**-**Rank test was used to compare survival curves. P values < 0.05 were considered statistically significant.

## Results

Ninty-one (59.5%) of the 153 patients were male, 62 (40.5%) were female, and the mean age was 41.65 ± 15.60 (range 18–82 years). The distribution of age and gender was not significantly different between groups (*P* = 0.56; Table 1). Frontal (58), temporal (48), parietal (24), occipital (10), infratentorial (7), and basal ganglia (6) were the areas where the tumors were located. Final histopathological diagnosis was LGG in 95 (62.1%) patients, and HGG in 58 (37.9%) patients. 84/95 (91.3%) of LGG patients and 50/58 (82.0%) of HGG patients had an appropriate initial diagnosis on the MRI obtained 72 h before surgery. In 92 patients with a preoperative radiological diagnosis of LGG, the histopathological diagnoses were 84 (91.3%) LGG and 8 (8.7%) HGG. The histopathological diagnoses in 61 patients having a preoperative radiological HGG diagnosis were 50 (82.0%) HGG and 11 (18.0%) LGG. The radiological-pathological concordance was statistically significant (*P* < 0.001).

**Table 1:** Study demographics

Parameters	Values	Io MRI group	Control group
Age (years)	Mean ± SD	40.58 ± 15.27	46.46 ± 16.44
	Minimum	18	19
	Maximum	82	70
Sex	Female Male	52 (41.6%) 73 (58.4%)	10 (35.7%) 18 (64.3%)
Side	Right Left	64 (51.2%) 61 (48.8%)	14 (50.0%) 14 (50.0%)
Location	Frontal Temporal Parietal Occipital Infratentorial Basal ganglia	48 (38.4%) 41 (32.8%) 21 (16.8%) 10 (8%) 3 (2.4%) 2 (1.6%)	10 (35.7%) 7 (25.0%) 3 (10.7%) - 4 (14.3%) 4 (14.3%)
WHO grade	LGG HGG	81 (64.8%) 44 (35.2%)	14 (50.0%) 14 (50.0%)
*WHO,* World Health Organization; *LGG,* low grade glioma; *HGG,* high grade glioma			

In the Io MRI group, the number of IDH1 mutant cases was 35 (28%) and the number of IDH1-wild cases was 43 (34.4%). IDH1 status was not studied in 47 cases (37.6%).

The frequency of GTR was significantly higher in patients with an initial radiological diagnosis of LGG (50/92) than those with HGG (23/61) (*P* = 0.044). In the final histopathological analysis of the excisional materials, it was seen that 52.6% (50/95) of patients with LGG and 39.7% (23/58) of patients with HGG achieved GTR (*P* = 0.119). The Ki-67 proliferation index value was > 15 in 52.9% of patients with HGG and 4.7% of patients with LGG (*P* < 0.0001).

Overall survival time according to glioma location were analyzed by Kaplan Meier curves using Log-Rank test. The shortest OS was seen in occipital gliomas [22.43 ± 4.8 months (95% CI: 4.79–48.3), *P* = 0.09] and the longest OS was seen in parietal gliomas [94.38 ± 7.03 months (95% CI: 80.58–108.17), *P* = 0.008].

## Io MRI group versus control group

Gross total resection was achieved in 73 patients: in 62 (84.9%) patients in the Io MRI group and in 11 (15.1%) patients in the control group. The EOR rates for both groups are presented in Table 2. Since optimal resection could not be achieved in 45 Io MRI applied cases, it was decided to continue the operation. In 20 of these patients, the tumor was completely removed. This raised the GTR rate from 33.6% to 49.6% in the Io MRI group.

**Table 2:** Distribution of tumor resection rates in both groups

EOR	Io MRI group (n = 125)	Control group ( n = 28)
GTR	62 (48.8%) LGG:4,HGG:58	11 (39.3%) LGG:4, HGG:7
STR	33 (26.4%) LGG:15, 18HGG	6 (21.4%) LGG:5, HGG:1
PR	26 (20.8%) LGG:14, HGG:12	8 (28.6%) LGG:4, HGG:4
Bx	4 (3.2%) LGG:4, HGG:0	3 (10.7%) LGG:1, HGG:2

*EOR,* extent of resection; *Io MRI*, intraoperative magnetic resonance imaging; *GTR*, gross total resection; *STR,* subtotal resection; *PR,* partial resection; *Bx,* biopsy

In 45 cases (36,0%) in the Io MRI group and 18 (64.3%) cases in the control group, the preoperative MRI images showed an irregular and heterogeneous pattern of contrast enhancement.

In 22.2% (10/45) of patients with a preoperative MRI diagnosis of HGG, and in 43.8% (35/80) of patients with LGG, the surgery was continued following Io MRI. In patients with radiological diagnosis of LGG, the decision to continue the operation was significantly higher than those with HGG (*P* = 0.02).

During the 10-year follow-up period, 12.8% (16/125) of the Io MRI cases and 57.1% (16/28) of the control cases developed relapse/progression. The relapse and progression rate in the Io MRI group was statistically significantly lower than in the control group. (*P* < 0.001). Table 3 presents the EOR status at the time of the first operation in cases that developed recurrence (and progression in tumor size was observed in cases with residual) during the 10-year follow-up.

**Table 3:** EOR properties of recurrent and progressive gliomas

Io MRI group (n = 16)			Control group (n = 16)	
HGG (n = 7)		LGG (n = 9)	HGG (n = 8)	LGG (n = 8)
GTR	2	5	5	3
STR	4	3	1	1
PR	1	1	1	3
Bx	-	-	1	1

*EOR,* extent of resection; *Io MRI*, intraoperative magnetic resonance imaging; *HGG,* high grade glioma; *LGG,* low grade glioma; *GTR, g*ross total resection; *STR,* subtotal resection; *PR,* partial resection; *Bx,* Biopsy

The median OS in the Io MRI group was 72.7 months (95% confidence interval [CI]: 64.2–81.2), while in the control group it was 134.4 months (95% CI: 107.5–161.4). There was no statistically significant difference between the two groups in terms of OS (*P* = 0.44). In the Io MRI group, the median PFS was 82 months (95% CI: 40.1–123.9), while in the control group it was 53 months (95% CI: 1.0–114.0). Patients in the Io MRI group had a statistically significant longer PFS compared to the control group (*P* = 0.004).

The median OS for LGGs in the Io MRI group was 85.3 months (95% confidence interval [CI]: 75.9–94.6), while in the control group it was 144.0 months (95% CI: 122.5–165.5), ( *P* = 0.28).

The median OS for HGGs in the Io MRI group was 41.4 months (95% confidence interval [CI]: 28.8–54.1), while in the control group it was 112.1 months (95% CI: 69.7–154.5), (*P* = 0.39).

In patients with GTR, the median OS was significantly longer (*P* = 0.01) than in patients with residual tumor tissue [109.84 months (95% CI: 97.8–121.8) and 102.7 months (95% CI: 82.3–123.0), respectively].

**Fig. 2:**
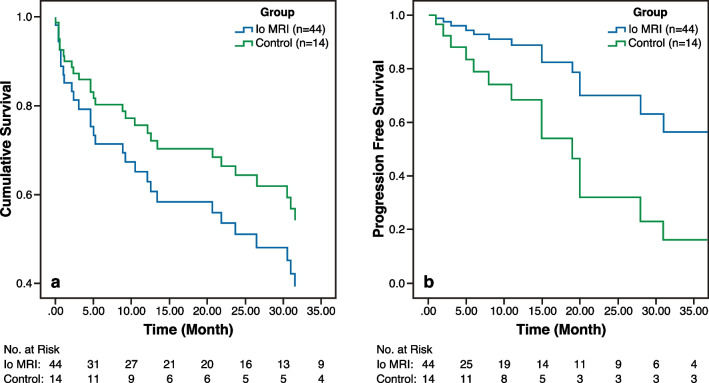
Kaplan–Meier curves show overall survival and progression-free survival rates in high grade glioma patients

**Fig. 3:**
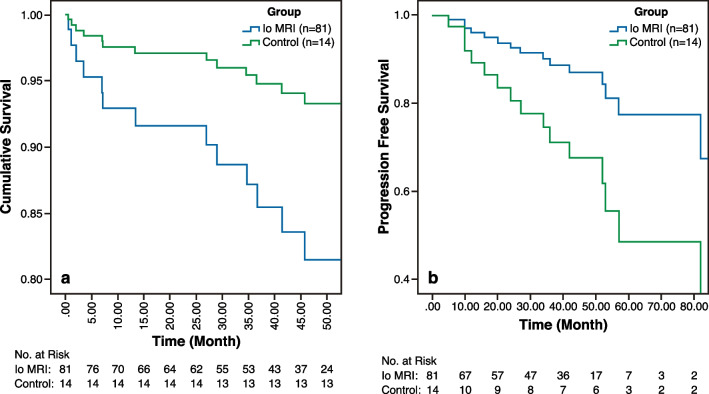
Kaplan–Meier curves showing the overall survival rate and progression-free survival rate in low grade glioma cases

**Fig. 4:**
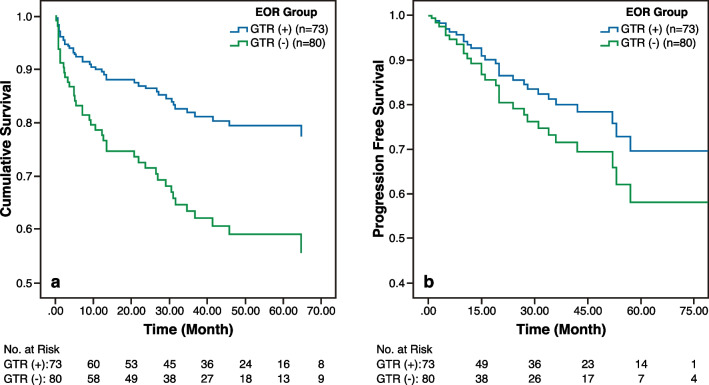
Kaplan–Meier curves showing the relationship between gross total resection and survival rates

**Fig. 5:**
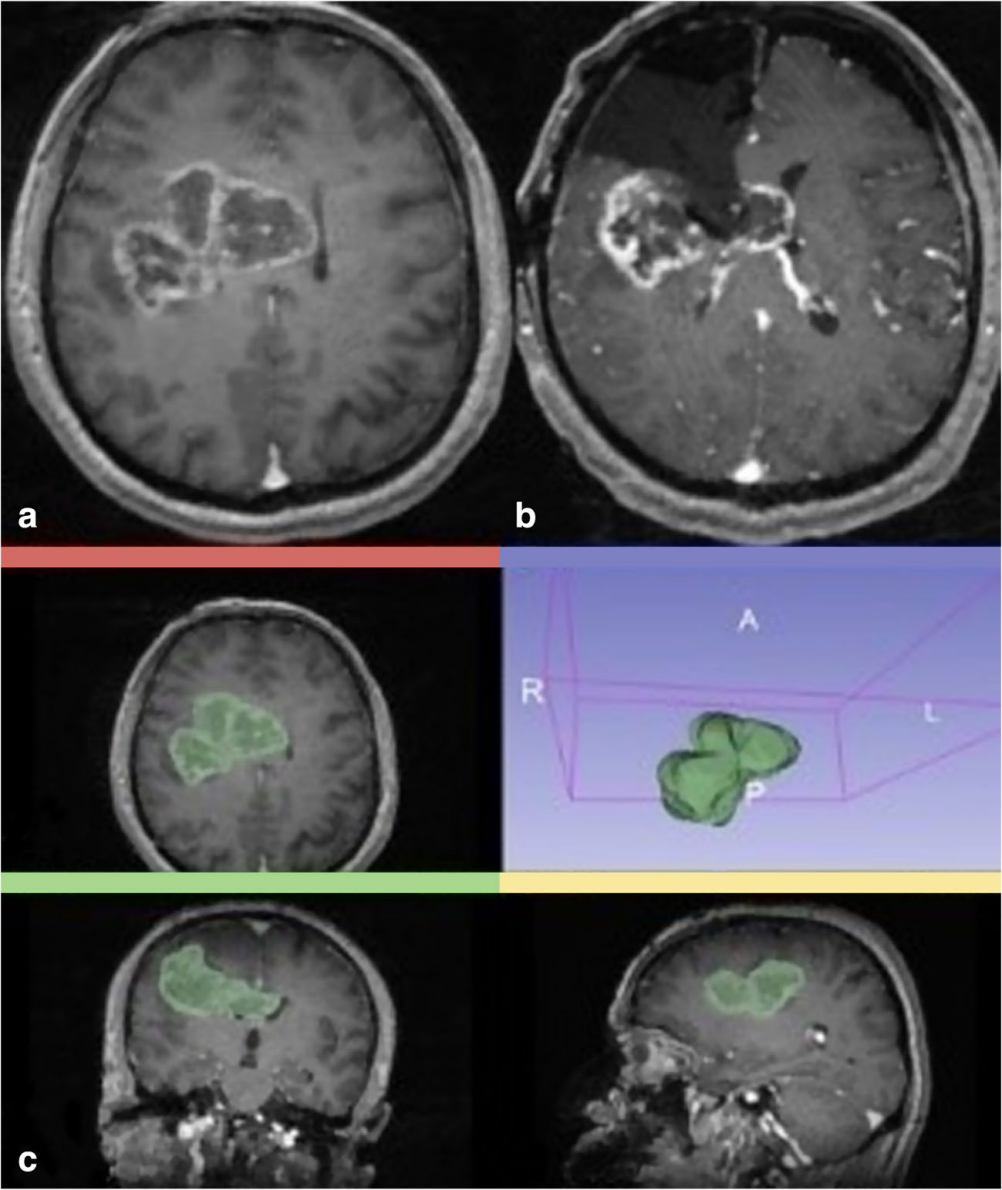
Illustrative Case: Preoperative postcontrast fat-saturated T1W axial brain MRI (**a**) shows right parietal high grade glioma with necrotic components. The mass is extending to the left hemisphere via the genu of the corpus callosum. Figure **b** shows T1W axial contrast-enhanced intraoperative MRI which is demonstrating partial resection of the mass. Preoperative volumetric measurement of the same mass is shown in Figure **c**

**Fig. 6:**
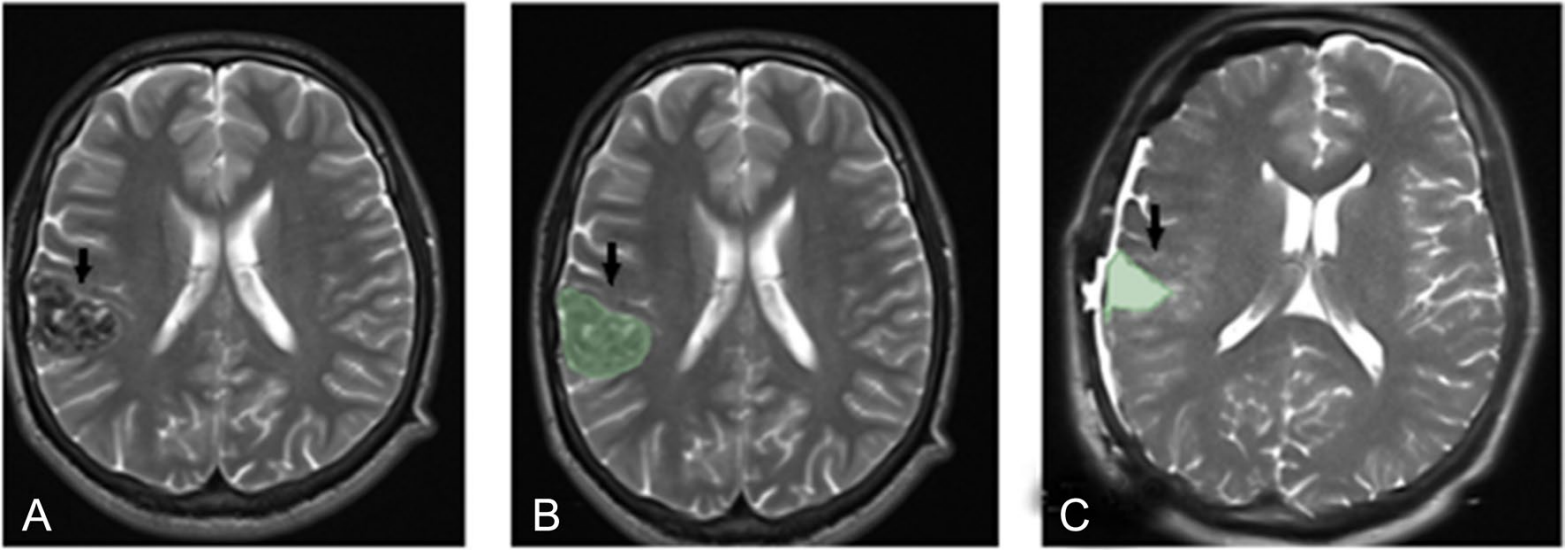
Preoperative axial T2W image (**a**, arrow), preoperative volumetric analysis image (**b**, arrow), and intraoperative axial T2W image after GTR (**c**, arrow) of the case with a histopathological diagnosis of ganglioglioma in the right temporal lobe

We also evaluated PFS in patients with HGG and LGG in Io MRI group vs control group. We found that the PFS was statistically significantly longer in the Io MRI group for HGG and LGG patients (*P* = 0.023 and *P* = 0.05, respectively). Figures 2, 3 and 4 depict OS and PFS rates for patients in each group using Kaplan–Meier curves (Figs. 5 and 6).

## Discussion

The use of high-field (3 T) magnetic resonance devices in intraoperative imaging allows for the acquisition of high spatial resolution images, permitting the surgeon to provide optimal tumor excision. However, due to the high sensitivity, it should taken into account that metal instruments such as non-ferromagnetic headrests and surgical equipment will cause sensitivity artifacts. In the study of Pamir et al., a 5**-**pin headrest**-**head coil combination specially designed for the system was used to solve this problem [12]. The 3 T MRI unit in our center is apart from the operation room. This design is suitable for both Io and outpatient imaging; its use for routine diagnostic purposes when Io imaging is not required reduces the financial cost.

In our study, 36% (45/125) of patients in the Io MRI group were returned to the operating room, increasing the resection rates. In a similar study by Bunyaratavej et al. that included glioma and non**-**glioma diagnoses, this rate was found to be 60% (24/40) [13]. These results show that the use of Io MRI in brain tumor surgery offers the possibility of an additional excision during the same surgical procedure in the event that the surgeon did not anticipate residual tumor before the operation.

Low**-**grade glial tumors are slow**-**growing, infiltrative masses with a median survival time of 5 to 7 years [14]. Since the response to chemotherapy and radiotherapy is poor [15], the widest possible resection of the tumor is targeted during the operation. Another reason for preferring the greatest acceptable resection is the potential of malignant degeneration of residual LGG [16]. In fact, some studies suggest that supratotal resection is associated with improved survival rates [14, 17]. Therefore, the surgeon may choose a more aggressive resection route when tumor features on the preoperative MRI are compatible with LGG. As expected, in our study, the EOR in lesions that were radiologically compatible with LGG was statistically significantly higher when compared to lesions considered to be HGG.

In the meta-analysis study by Li et al., the effect of using Io MRI on the resection rate and survival was evaluated, and it was concluded that the PFS was longer in the groups that underwent Io MRI compared to conventional neuronavigation (*P* = 0.012); however, OS rates did not differ between groups *(p* = 0.799) [6]. They also reported that Io MRI significantly improved PFS and EOR, especially in HGG patients[6]. In a study involving 14 patients diagnosed with glioblastoma, Pietel et al. revealed no significant difference between the median survival rates of patients who underwent Io MRI and those who did not (*P* = 0.68) [18]. Roder et al. noted that the use of Io MRI in 117 cases of glioblastoma raised the 6-month PFS rate from 32 to 45%, although no statistically significant data could be obtained (*P* = 0.131) [19]. They related this result to the use of varied adjuvant treatment protocols on patients and the limited number of cases. In the comprehensive meta-analysis published by Caras et al., techniques such as Io MRI, diffusion tensor imaging, and functional MRI were investigated in glioma surgery [20]. It was concluded that the aforementioned intraoperative imaging methods significantly increased OS and GTR compared to standard neuronavigation (35.0 months vs. 14.4 months, *P* < 0.001 for both parameters).

In studies investigating the effect of tumor localisation on OS, it has been reported that periventricular extension and eloquent area involvement decrease OS. In the study of Liu et al., the decrease in OS in occipitotemporal glioma cases is supported by the results of our current study [21]. It was emphasized that the distance between the center of the third ventricle and the edge of the contrast-enhancing tumor affects the survival time [22]. This distance is shorter in centrally located tumors. Centrally located gliomas can extend more easily through deep white matter tracts, making excision difficult. This may increase the possibility of surgical sequelae and decrease OS.

PFS in the Io MRI group was significantly longer than in the control group in the current study. The absence of a statistically significant difference in OS rates may be due to the use of adjuvant therapy, which varies according to histopathological subtype and molecular profile. In addition, the fact that the HGG/LGG distribution is not homogeneous between the groups can be considered another reason.

Our study has several limitations. The first is the retrospective nature of research design. The second is the limited number of control patients due to presence of an Io imaging system in our institution. The third limitation factor is that the World Health Organization classification of brain neoplasms was updated in May 2021, and the majority of our patients’ histopathological diagnoses were recorded according to the 4th edition published in 2016. According to the last classification, CNS WHO grades of isocitrate**-**dehydrogenase (IDH) mutant astrocytomas range from 2 to 4; IDH mutant oligodendrogliomas with 1p/19q deletion have both CNS WHO grade 2 and 3 types [23]. Because of this current information, the aforementioned neoplasms are not called LGG without knowing their molecular panel.

## Conclusion

Recent research has demonstrated the benefits of Io MRI in the excision of various intracranial tumors such as glioma, meningioma, pituitary neoplasms, schwannoma [24, 25]. Gliomas are the group of tumors for which intraoperative imaging is most needed. In this study, the convenience provided by the high-field MRI device was explored and proven both in reducing the tumor burden, increasing the PFS, and providing the surgeon with a maximal and optimal resection in the first operation. Future study will require larger sample sizes, the inclusion of histopathological diagnoses based on molecular/genetic profiles, and more homogenous case groups.

## Data Availability

No datasets were generated or analysed during the current study.
